# Research on a high-sensitivity asymmetric metamaterial structure and its application as microwave sensor

**DOI:** 10.1038/s41598-022-05255-2

**Published:** 2022-01-24

**Authors:** Yunhao Cao, Cunjun Ruan, Kanglong Chen, Xingyun Zhang

**Affiliations:** 1 School of Electronic and Information Engineering, Beihang University, Beijing, 100191 China; 2 Beijing Key Laboratory for Microwave Sensing and Security Applications, Beihang University, Beijing, 100191 China

**Keywords:** Sensors and biosensors, Characterization and analytical techniques

## Abstract

In this paper, an Asymmetric Electric Split-Ring Resonator (AESRR) metamaterial structure is proposed to explore the interaction between metamaterials and electromagnetic waves with the influence of Fano resonance on electromagnetic properties. With the symmetry of basic electric Split-Ring Resonator (eSRR) being broken, a new Fano resonant peak appears at around 11.575 GHz and this peak is very sensitive to the dielectric environment. Based on the proposed high sensitivity of AESRR, a microwave sensor based on a 13 × 13 arrays of AESRR was designed and verified using printed circuit board (PCB) technology. T-shape channel was integrated to the sensor by grooving in the FR-4 substrate which improved the integration and provided the feasibility of liquids detection. Seven organic liquids and four dielectric substrates are measured by this sensor. The measured results show the transmission frequency shifts from 11.575 to 11.150 GHz as the liquid samples permittivity changes from 1 to 7 and the transmission frequency shifts from 11.575 to 8.260 GHz as the solid substrates permittivity changes from 1 to 9. The results have proven the improved sensitivity and the larger frequency shift **∆*f*** on material under test (MUTs) compared with the conventional reported sensor. The relative permittivity of liquid samples and solid samples can be obtained by establishing approximate models in CST, respectively. Two transcendental equations derived from measured results are proposed to predict the relative permittivity of liquid samples and solids samples. The accuracy and reliability of measured results and predicted results are numerically verified by comparing them with literature values. Thus, the proposed sensor has many advantages, such as low-cost, high-sensitivity, high-robustness, and extensive detecting range, which provided a great potential to be implemented in a lab-on-a-chip sensor system in the future.

## Introduction

Metamaterials are artificially made electromagnetic materials composed of sub-wavelength resonant elements, which can manipulate electromagnetic wave beams and exhibit some exotic electromagnetic properties by manipulating their structural geometry and arrangement^1,2^. Metamaterials have many unique electromagnetic properties that are not found in natural materials, such as negative dielectric constant and negative permeability, etc. Moreover, specific metamaterial structures have the electromagnetic property that is very sensitive to the change of dielectric environment^3–6^. Microwave sensors have many advantages such as low fabrication and measurement cost, CMOS compatibility, design flexibility, and real-time response. The high sensitivity of metamaterials and the advantages of microwave sensors allow microwave metamaterial-inspired sensors to be widely used in various fields, such as chemical, biosensing, substrate detection, and microfluidic systems^7–12^.

Recently, many new and improved microwave sensor based on meta-atom structure were developed to distinguish and detect different liquids. In 2013, a microfluidic sensor implemented from a single split-ring resonator (SRR) was proposed for the dielectric characterization of liquid samples^3^. In 2014, a new microwave device which was composed of a microstrip coupled complementary split-ring resonator (CSRR) was proposed in reference^4^ as a microfluidic sensor. The sensor can identify water–ethanol mixtures of different concentrations and determine their complex permittivity. In 2017, a meta-atom split-ring resonator (SRR) with a microfluidic channel positioned in the gaps was proposed to distinguish and detect different water–ethanol or water–methanol mixtures. As a passive microwave device without additional physical connections, the excitation source of the sensor is the antenna rather than the conventional microstrip line^13^. In 2019, A microwave sensor using a Complementary Circular Spiral Resonator (CCSR) was designed for identifying different liquid samples and determining their dielectric constants by dropping the liquids on the sensitive area^5^. In 2020, a microwave sensor with a planar circular complementary spilt-ring resonators (CSRRs) was proposed and fabricated by using printed circuit board (PCB) technology^8^. The sensor with tube inside the PCB substrate can measured different liquids and estimate their primitivity based on the measured ${S}_{21}$ results. Many other microfluidic sensors based on different meta-atom structures^6–8^ were reported to distinguish different liquids and determine their permittivity, such as water, hexane, chloroform, water–ethanol or water–methanol mixtures.

The response of a material to electric signal depends on the permittivity of materials. In the field of electronics, dielectric constant is an important electromagnetic characteristic of materials. Recently, many new microwave sensors based on basic metamaterial structure have been proposed and used for distinguishing different solids materials and detecting their permittivity.

In 2012, Boybay et al. proposed a microwave method for dielectric characterization of planar materials by using complementary circular split-ring resonators (CSRRs) working at a 0.8–1.3 GHz band^11^. In 2014, A complementary square split-ring resonators (CSRRs) sensor, operating at 1.8 GHz to 2.8 GHz, was proposed and fabricated for distinguishing different solid materials and measuring the dielectric constants and loss tangents of materials^12^. In 2018, A microwave sensor based on a single ring resonator structure was used to identify not only the relative permittivity but also the thickness of different materials attached to the sensor^10^. And a parabolic equation was proposed to predict the relative permittivity of material based on the measured resonant frequency. In 2019, A microwave sensor, excited by microstrip line and based on the complementary circular spiral resonator (CCSR), was reported for distinguishing and nondestructively estimating different dielectric substrates^9^. And a transcendental equation was established to estimated the relative permittivity of unknown materials based on the measured resonant frequency.

Among the aforementioned typical microwave characterization devices, all of them are based on the most basic metamaterial structures and are suitable for either liquid detection only or solid detection only. And microfluidic sensors^4–8^ can only distinguish some liquids with high permittivity such as water–ethanol mixtures of different concentrations of which the dielectric constants vary greatly. The sensor for material characterization of solids^9–12^ can distinguish different substrate materials with tiny frequency shift ***∆f***, so there is still a lot of room for improvement in sensitivity. Meanwhile, most of the reported microwave sensors composed by meta-atom structure are easily influenced by the surroundings, leading to the low stability of the sensor. Therefore, the current microwave sensors based on metamaterials/left-handed materials still have a lot of room for improvement in sensitivity and stability.

In this paper, to research the interaction between metamaterials and electromagnetic waves and the influence of Fano resonance on electromagnetic properties of metamaterials, an Asymmetric Electric Split-Ring Resonator (AESRR) is proposed based on the basic electric Split-Ring Resonator (eSRR) metamaterial structure and the Fano resonance. The simulated results show that there appears a novel resonance peak at around 10.575 GHz and this new Fano resonance peak is very sensitive to the change of the dielectric surroundings. To verified the high sensitivity of AESRR and make a concrete application, a microwave metamaterial-inspired sensor based on a 13 × 13 arrays of AESRR is designed for liquids and solids detection. The AESRR metamaterial structure is used in place of eSRR structure^14^ to provide a novel resonance peak and increase the sensitivity of the sensor. The T-shape channel covering the sensitive region of sensor was integrated to sensor by grooving in the substrate which greatly improved the integration of the microwave passive device. This sensor was fabricated by employing PCB fabrication technology and has been verified to have the ability to distinguish seven organic liquids and four common dielectric substrates based on their different frequency shift ***∆f***. The dielectric constant of MUTs can also be obtained by using relatively accurate simulation models, which were built in CST according to actual measurement environment. Moreover, two transcendental equations are proposed to predict the relative permittivity of liquid samples and solid materials based on the measured resonant frequency, respectively. The proposed sensor can measure not only liquids but also solids and it offers a high robust, high sensitivity, high integration, low fabrication cost and low measurement cost which is promising to be implemented in a lab-on-a-chip system in the future.

On the basis of the previous work^15^, this paper has optimized the substrate thickness and channel for higher sensitivity, wider permittivity range studied and further application. Design of AESRR structure, performance analysis of the whole sensor and sensor fabrication is explained in “Sensor design and fabrication” section. Measurement and transcendental equation for five liquids is performed in “Measured results of different liquids with low dielectric constant” section. Measurement and transcendental equation for four solid materials is shown in “Measurement for solid dielectric substrates” section. The sensor performance compared to some conventional microwave sensor is discussed in “Performance comparison” section and the research is concluded in “Conclusion” section.

## Sensor design and fabrication

### Metamaterials design and sensor design

**Figure 1:**
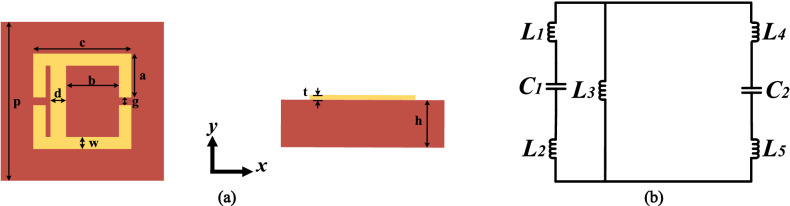
(**a**) Schematic of the unit asymmetric eSRR (AESRR) structure. (**b**) The equivalent circuit of AESRR.

Figure 1a shows the schematic of asymmetric eSRR (AESRR) structure, the primary component of the proposed metamaterial-inspired sensor. AESRR is chosen as the fundamental building block of metamaterials because of its simplicity and sensitivity to the change of permittivity environment. The material of AESRR is copper (pure) with electrical conductivity of 5.96 × 10^7^ s/m and the substrate is FR-4 (lossy) with a dielectric constant of 4.4. The dimensions of the AESRR metamaterial structure were given in Table 1.

**Table 1:** Structure parameters of AESRR.

Parameter	Value [mm]
*a*	2.75
*b*	3.30
*c*	6.00
*d*	0.90
*g*	0.50
*p*	10.00
*w*	0.75
*t*	0.03
*h*	1.00

Figure 1b shows the equivalent circuit model of AESRR metamaterial structure. In the equivalent circuit, ${L}_{1}$, ${L}_{2}$, ${L}_{3}$, ${L}_{4}$, and ${L}_{5}$ represent the equivalent inductances of the metal arms in the corresponding position, respectively. ${C}_{1}$ and ${C}_{2}$ are the equivalent capacitances of the gaps of AESRR. Among the circuit element, the values of inductance ${L}_{1}$-${L}_{5}$ related to the sensor itself are determined by the structural parameters and the composition materials of the sensor. Equivalent circuit model^4^ concludes that the equivalent capacitance of the gaps of sensor is determined by the capacitive effects of sensor itself and the effect of MUTs. According to the equivalent circuit model, the equivalent capacitance ${C}_{1}$ and ${C}_{2}$ can be expressed as:

1 $${C}_{1}={{C}_{0}}^{{{\prime}}}+ {({\varepsilon }_{sam}{C}_{c})}^{{{\prime}}} \; \text{and} \; {C}_{2}={{ C}_{0}}^{{{\prime}}{{\prime}}}+ {({\varepsilon }_{sam}{C}_{c})}^{{{\prime}}{{\prime}}}$$

where ${{C}_{0}}^{{{\prime}}}$ and ${{C}_{0}}^{{{\prime}}{{\prime}}}$ model the capacitive effects on both sides of the gaps, which are determined by the dielectric substrate, channels, and surrounding space of the sensor itself. The term ${({\varepsilon }_{sam}{C}_{c})}^{{{\prime}}}$ and ${({\varepsilon }_{sam}{C}_{c})}^{{{\prime}}{{\prime}}}$ describe the dielectric contribution from the load MUTs with ${C}_{C}$ being the capacitance of an empty channel and ${\varepsilon }_{sam}$ being the permittivity of MUTs. The value of the effective capacitance ${C}_{g}$, the total equivalent capacitance of the sensor including ${C}_{1}$ and ${C}_{2}$, is influenced by the dielectric materials around the gaps and can be approximately expressed as^13^:

2 $${C}_{g}={C}_{1}+{C}_{2}={C}_{0}+{\varepsilon }_{sam}{C}_{c}$$

As mentioned above, ${C}_{0}$ models the total capacitive effects determined by the sensor itself and the term ${\varepsilon }_{sam}{C}_{c}$ describes the total dielectric contribution from the load MUTs.

The resonant frequency (${f}_{0}$) of the sensor can be defined as:

3 $${f}_{0}=\frac{1}{2\pi \sqrt{L({C}_{g})}}$$

where L represents the total equivalent inductance of the AESRR structure. From (1)–(3), the resonant frequency can be functions of the load MUTs permittivity as (4) shows:

4 $${f}_{0}={F}_{1}({\varepsilon }_{sam})$$

This indicates that the resonant frequency of the sensor will be influenced by the permittivity of the load MUTs^16^. Therefore, the dielectric constant of an unknown MUTs can be determined simply by measuring the different transmission resonant frequencies of sensor due to the interaction with different MUTs.

All the simulation in this paper was calculated in the *periodic structure* workflows of *MW & RF & Optical* application in CST^17,18^. About the simulation model, the meth type is *Tetrahedral* mesh, the mesh generation is adopted the Adaptive *Tetrahedral Mesh Refinement*, and the broadband sweep is *general purpose*. As for the boundary conditions, the simulation model is a periodic structure, each metamaterial unit structure is surrounded by eight other metamaterial units, so the X and Y directions was set to “*unit cell*” and the Z directions of the model were set to “*open (add space)*”. The excitation of the simulation model is plane wave, electromagnetic waves travel along the Z axis and through metamaterials, the electric field is parallel to the X axis (middle metal arm).

**Figure 2:**
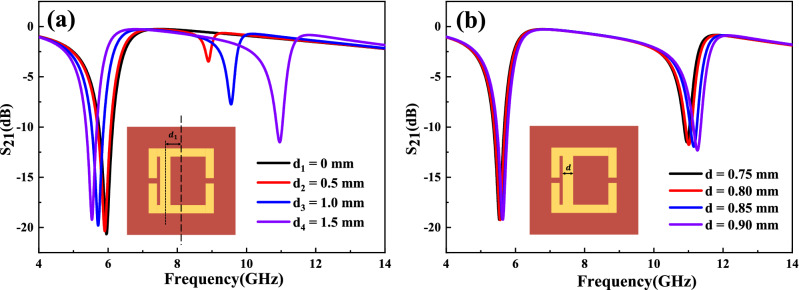
Design and optimization of AESRR. (**a**) Optimization of the shift of middle metal arm (${d}_{1}$). (**b**) Optimization of the width of middle metal arm ($d$).

The Fano resonance, discovered by Ugo Fano in 1961, has been described as the interference between continuum of states (the scattered states) and quasi-bound states (resonant states)^19^. Sekar et al. concluded that introducing Fano resonance to the metamaterial structure is an efficient way to generate a new resonance peak improving the sensitivity of the sensor^16^. Figure 2 show the design and optimization of AESRR metamaterials structure. To obtain a novel sensitivity resonance peak, we try to offset the middle metal arm by some distance (${d}_{1}$). The simulated results in Fig. 2a show that there is a new resonance peak with middle metal offsetting a distance ${d}_{1}$. And the larger the offset, the more obvious the resonance peak. To ensure machinability, the shift ${d}_{1}$ was finally determined to be 1.5 mm. Meanwhile, we optimized and increased the width of middle metal arm to make the electric field stronger. The simulated results in Fig. 2b show that as the width of middle metal arm $d$ increases, the amplitude of the novel peak becomes larger which is useful for practical measurement.

**Figure 3:**
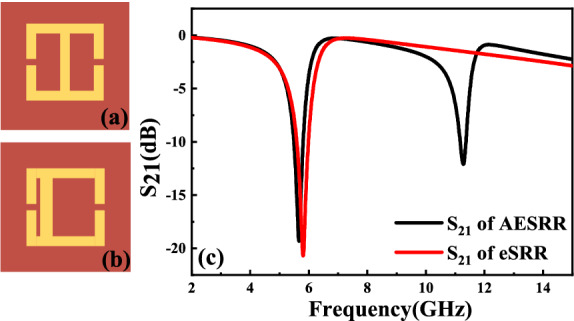
(**a**) The symmetric eSRR metamaterial structure. (**b**) The asymmetric eSRR (AESRR) metamaterial structure. (**c**) Simulated ${S}_{21}$ of these two structures.

The basic eSRR metamaterial structure is shown in Fig. 3a. To achieve higher sensitivity, the asymmetric eSRR (AESRR) structure is proposed based on the Fano resonance, shown in Fig. 3b. The Fano resonance is generally caused by asymmetric metamaterial structures^16^. As Fig. 3c shows, there appears a novel Fano peak at around 11.30 GHz with the symmetry of eSRR destroyed.

**Figure 4:**
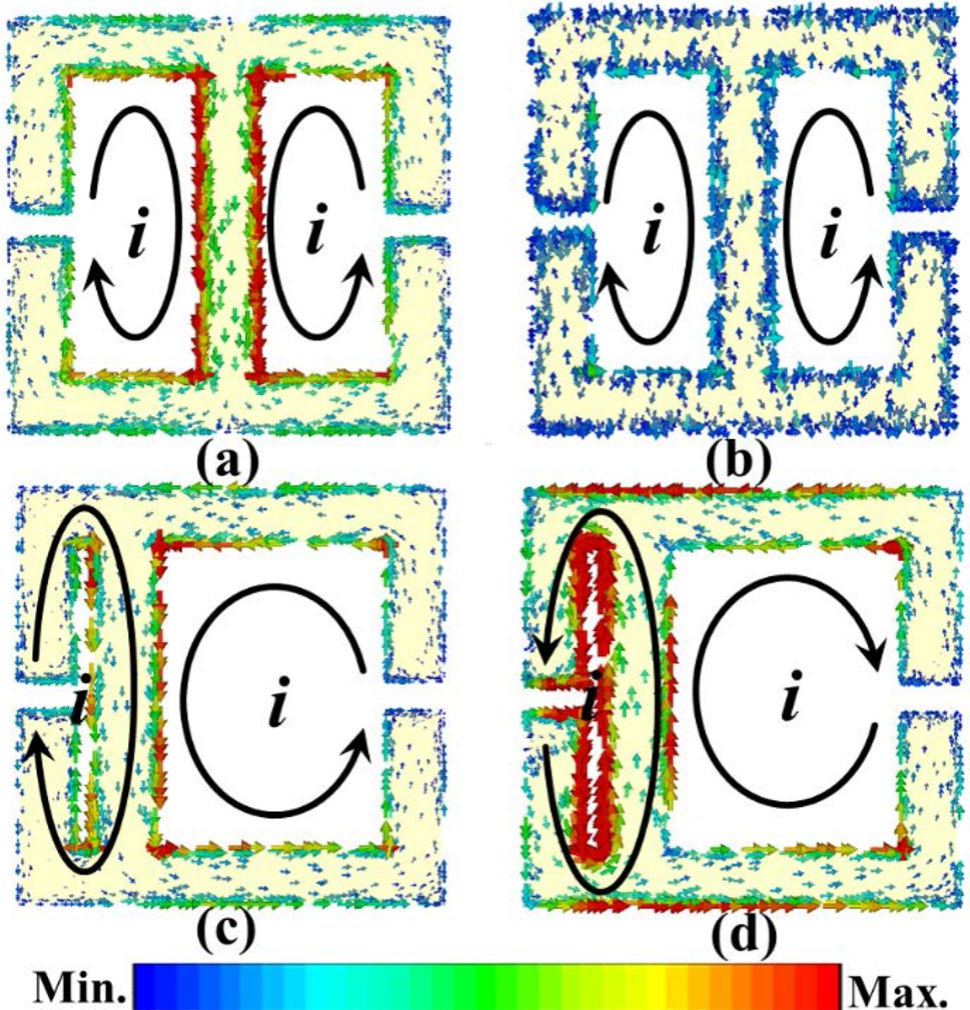
(**a**) Surface current simulation in eSRR at transmission resonance peak at 5.81 GHz. (**b**) Surface current simulation in eSRR at 11.28 GHz. (**c**) Surface current simulation in AESRR at transmission resonance peak at 5.67 GHz. (**d**) Surface current simulation in AESRR at transmission resonance peak at 11.28 GHz.

**Figure 5:**
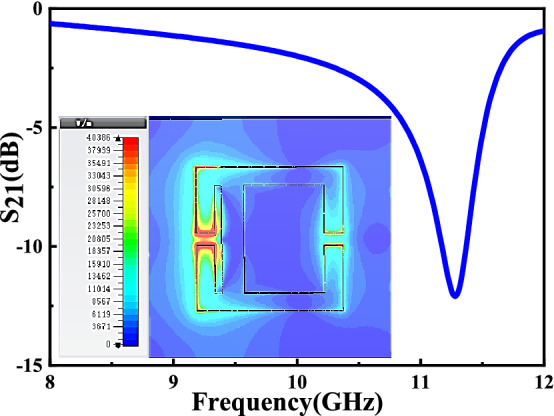
Simulated ${S}_{21}$ transmission resonance of the proposed sensor without T-shape channel in CST Studio Suite and Electric field distribution at resonant frequency 11.28 GHz.

**Figure 6:**
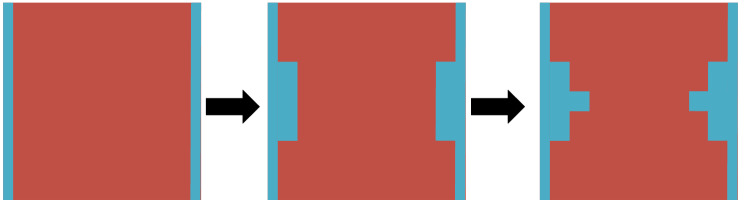
Design and optimization of T-shape microfluidic channel.

Figure 4 shows surface current simulation in eSRR and AESRR at different frequency. As Fig. 4a shows, the currents in the two equal metal wire arms of eSRR oscillate in phase and interfere constructively^7^, which generates a resonance peak at 5.81 GHz. Compare to the eSRR, the two current loops in AESRR differ with the symmetry broken, leading to a strong coupling between them. Generally speaking, the longer the current path is, the lower the frequency of resonance peak is; The shorter the current path, the higher the resonant frequency. In Fig. 4c, The right current loop is slightly stronger than the left current loop. In Fig. 4d, The left current loop is obviously stronger than the left current loop. The resonant peak of AESRR at 5.67 GHz is from the large current path on the right and the resonant peak of AESRR at 11.28 GHz is from the small current path on the right. By comparing Fig. 4b,d, the current loop in AESRR is stronger and the current difference between the two loops in AESRR is larger, which created a strong coupling and generated a new resonance peak at 11.28 GHz. And the electric field distribution of AESRR at transmission resonance peak at 11.28 GHz is shown in Fig. 4. Electric field distribution embedded in Fig. 5 tells us that a strong electric field establishes between gaps, especially the left one. To ensure the performance of the sensor, the channel should cover the sensitive areas. Whereas the width of the gap **(g)** increases the difficulty of the microfluidic channel processing and integration. Considering the integration difficulty and processing cost, finally, we decide to process and integrate the T-shape microfluidic by grooving in the FR-4 substrate. Another consideration was a lab-on-chip system implementation, which is convenient with microfluidic channel in substrate. The design and optimization of T-shape microfluidic channel was shown in Fig. 6 shows and the specific parameters of channel are described in the following sections.

**Figure 7:**
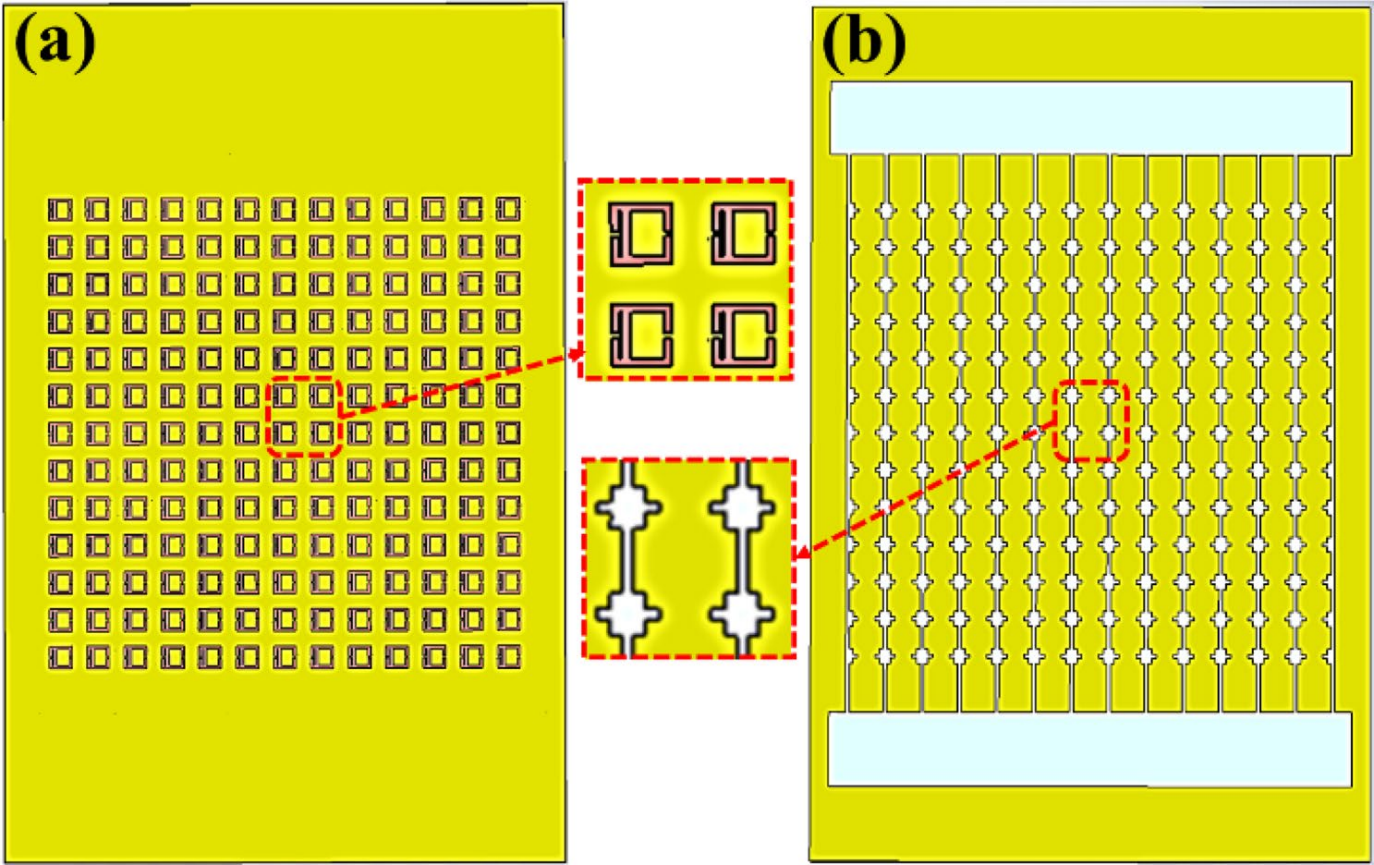
(**a**) the 13 × 13 periodic arrays AESRR structure of the integrated AESRR metamaterial-inspired sensor. (**b**) the schematic of the whole microfluidic channel.

As Fig. 7 shows, a metamaterial-inspired sensor based on a 13 × 13 AESRR arrays structure has been designed to enable the feasibility and the accuracy of the measured results. Figure 7a shows a 13 × 13 AESRR arrays which is large enough to cover the radiation range of the antenna to ensure the reliability of the measurement. Figure 7b is the schematic of the whole microfluidic channel. As Fig. 7b shows, we also designed two square grooves on the both edges of the microfluidic channel so that it is convenient for us to make the liquid samples in the square grooves fill in the channel with the help of the gravity and fluidity of liquid samples.

**Figure 8:**
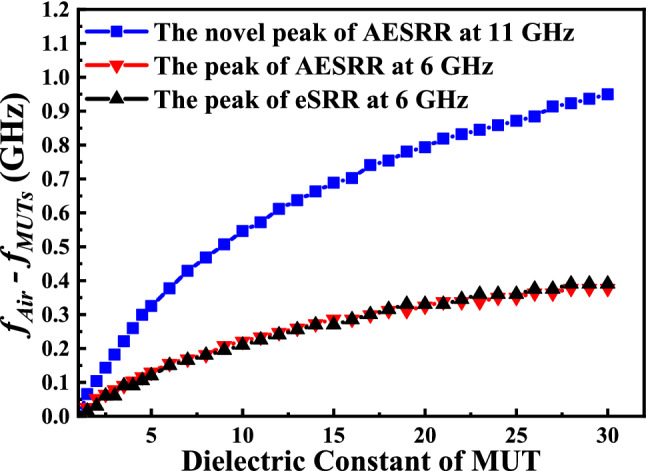
Simulated results of different resonance peaks (the peaks of AESRR at around 6 GHz and 11 GHz; the peaks of eSRR at around 6 GHz) with channel filled with different dielectric materials.

In order to verify the performance of the proposed sensor and compare the sensitivity of different resonance peaks, sensors based on different metamaterial structures was analyzed in the CST^17,18^. By changing the dielectric constant of MUTs in the channel, different resonance peaks have different frequency shift $|\Delta f|$. Figure 8 clearly illustrates that the sensitivity of the peak of AESRR is much better than that of the other two resonance peaks. The simulated frequency shift $|\Delta f|$ shows that the resonance peak of AESRR at around 6 GHz and the resonance peak of eSRR at around 6 GHz are insensitive to small changes in the dielectric environment unless the changes in the dielectric environment are large enough. At the same time, the resonant peak of AESRR at around 11 GHz has a large $|\Delta f|$ even for the slight changes of dielectric environment. Based on the simulated results, the resonance peak at around 11 GHz was selected for measuring different MUTs with slight dielectric change.

### Sensor fabrication and measurement setup

**Figure 9:**
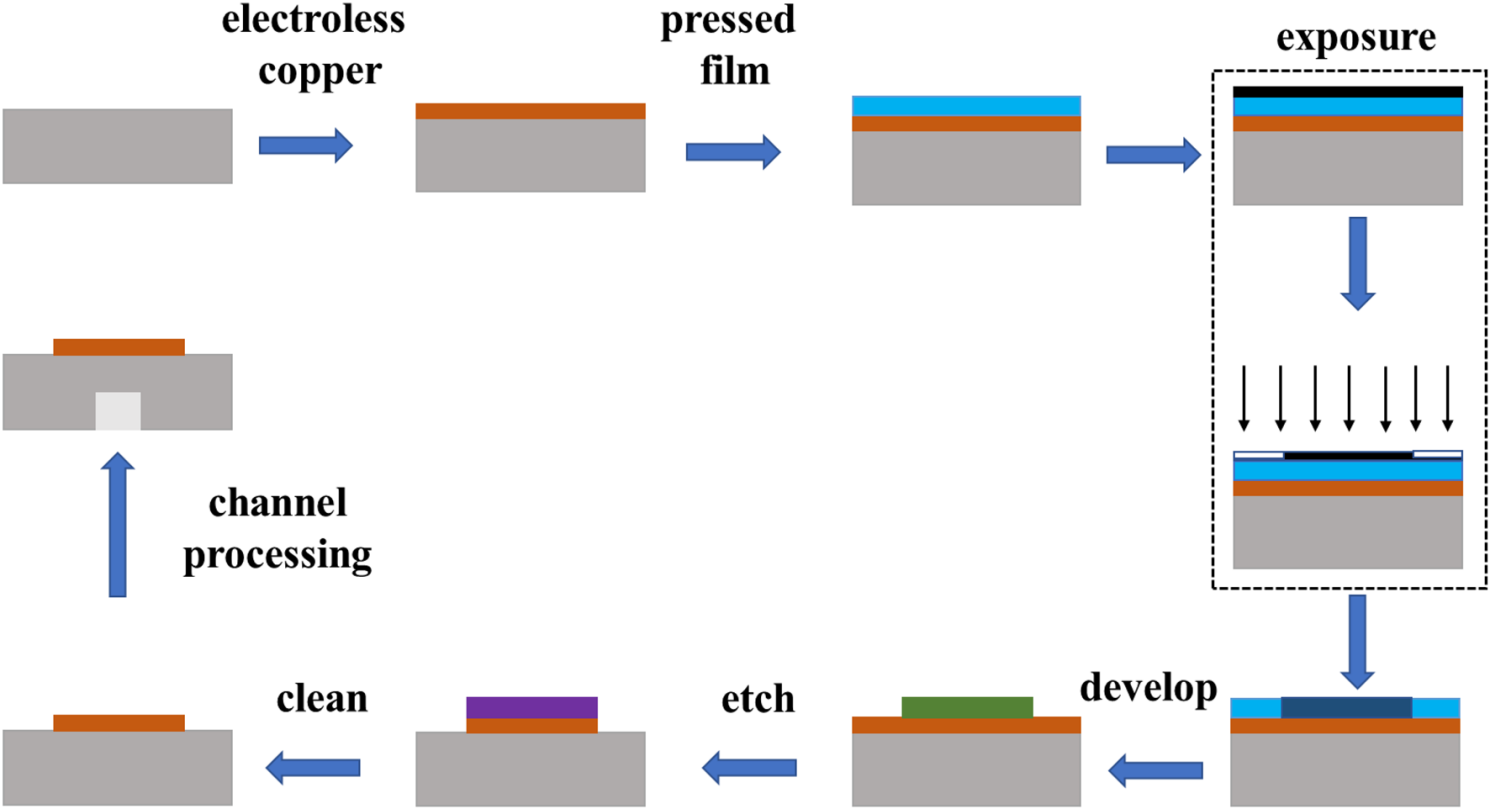
The simple schematic diagram of the manufacturing process.

**Figure 10:**
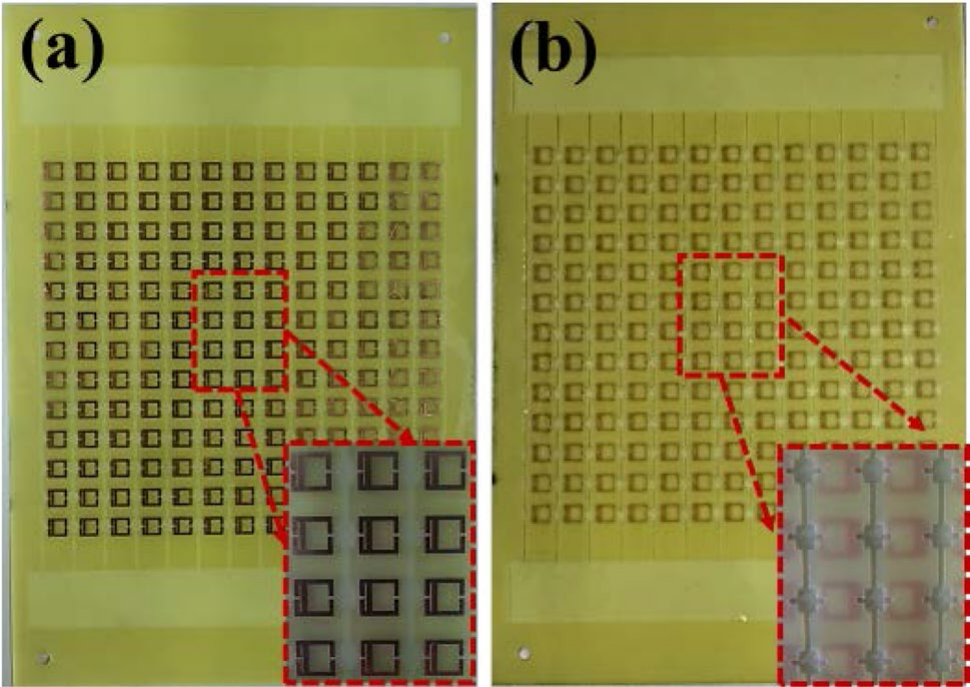
(**a**) Overall photograph and partial enlarge view of the 13 × 13 periodic arrays AESRR structure. (**b**) Overall photograph and partial enlarge view of the whole microfluidic channel.

We fabricated the sensor based on the AESRR by employing the PCB fabrication technology. The simple schematic diagram of the manufacturing process is as Fig. 9 shows. There are several key steps in the whole process: board cut—plated through hole (PTH)—pressed film—exposure—develop—etch—clean—channel processing, then we get the microwave sensor. Considering the characteristic of the patch antenna and in order to ensure the accuracy of the measurement result, a 13 × 13 AESRR arrays plant was fabricated on the FR-4 substrate, with a relative permittivity of 4.4 and was 13 cm × 25 cm in size, shown in Fig. 10. This sensor is a kind of passive microwave device, and has the advantage being high-robust, reusable, real-time and high-sensitivity.

**Figure 11:**
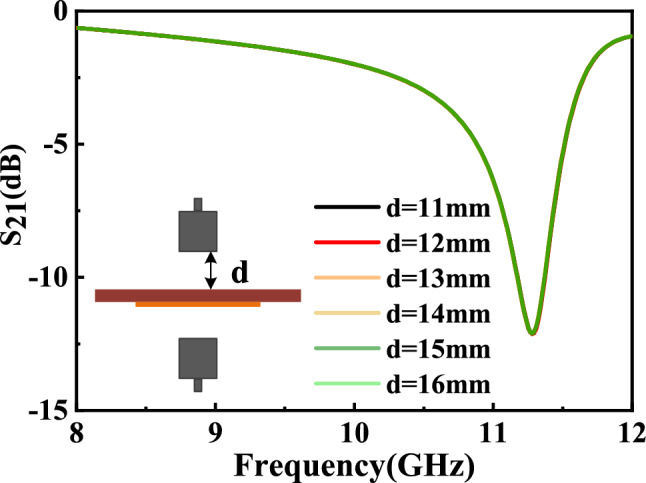
Simulation results of the influence of the distance between the antenna and the sensor on the measured results.

In the simulation software (CST) the distance between transmitting antenna and sensor must greater than 10.2 mm which is determined by substrate thickness (1 mm) and periodic structure characteristics. Figure 11 shows the effect of the distance on the measured results. It’s not hard to find the distance between transmitting antenna and sensor has very little effect on the measured results. Considering the attenuation of the antenna is severe when the distance is large, so we decided to keep the distance between 1.2 and 1.6 mm.

**Figure 12:**
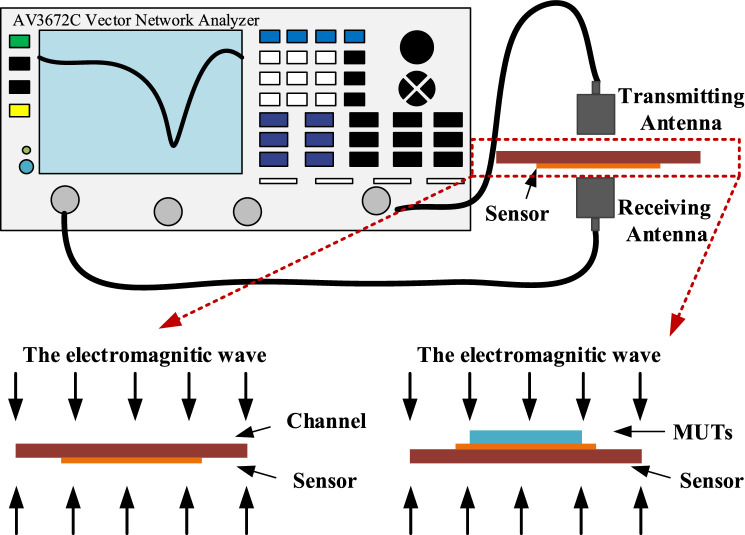
Deployment of the proposed microwave sensing system for the dielectric characterization of organic liquids and solid dielectric substrates with low dielectric constant.

The schematic diagram of the developed microwave sensor for dielectric characterization and its deployment are shown in Fig. 12. All the experiments were carried out at a room temperature of 25 °C. In our measurement, signal is generated by vector network analyzer (AV3672C, 10 MHz–43.5 GHz), and a pair of patch antennas are used to transmit and receive signals, shown in Fig. 12.

### Device characterization

**Figure 13:**
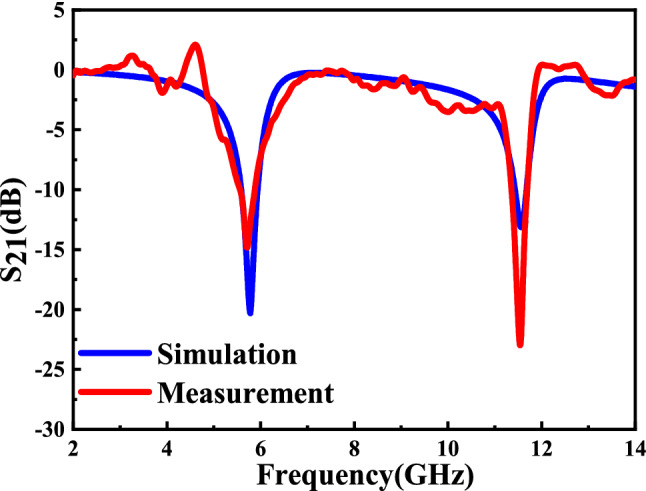
Simulated and measured transmission response ${S}_{21}$ of the sensor without MUTs.

To test whether electromagnetic waves reflected from the ground actually affect the measurement results and eliminate the influence of the surrounding environment on the measurement results, we put absorbent materials under it to test it experimentally. When the microwave sensor is not placed between the antenna, the measured results show that there is no any resonant peak appeared. Once we placed the sensor (without sample put on it) between the antenna without any change, the measured results show there are two distinct resonant peaks, which show the resonant peaks are completely caused by our AESRR of the sensor. Meanwhile, in terms of measured resonant frequencies, the results show that electromagnetic waves reflected from the ground have little effect on the results and the ground reflection can be ignored. The measured and simulated results of ${S}_{21}$ transmission coefficient of the sensor is shown in Fig. 13. It’s obvious that the measured results are basically consistent with the simulation results. When the measurement platform and the sensor without MUT was set up according to the deployment in Fig. 12, about 15 times measurement was carried out to make sure the measured results are reliable, and the measured results indicate that the sensitive peak is stabilized at 11.575 GHz. Detailed data of the simulated and measured results are given in Table 2. The difference of amplitude between simulated and measured results is mainly due to the characteristics of patch antenna, fabrication tolerance, conductor, dielectric and radiation losses. Considering that the proposed device distinguishes different MUTs based on the shift of the resonant frequency, the measured results indicate that the device conforms to the design and can be used as a sensor.

**Table 2:** Measured and simulated results of the microwave sensor without MUTs.

Results	${f}_{1}$ (GHz)	${Notch \; depth}_{1}$, dB	${f}_{2}$ (GHz)	${Notch \; depth}_{2}$, dB
Simulated	5.760	− 20.2	11.570	− 13.2
Measured	5.720	− 16.1	11.575 ± 0.01	− 25.4

## Measured results of different liquids with low dielectric constant

**Figure 14:**
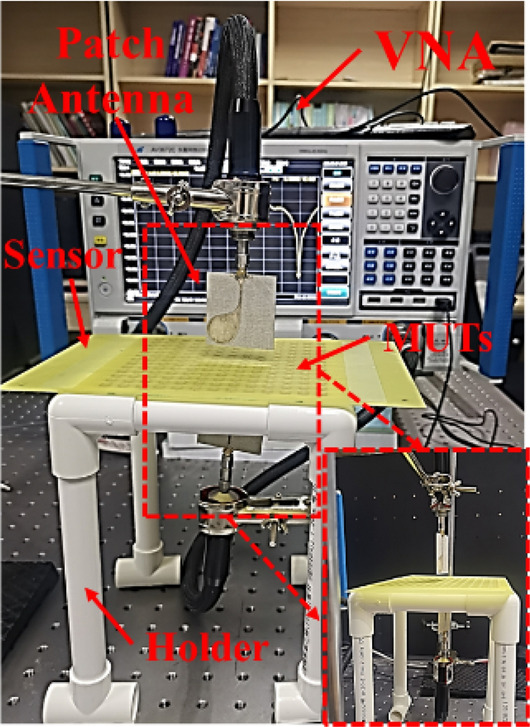
Photograph of the experiment platform for measuring different liquid samples.

**Figure 15:**
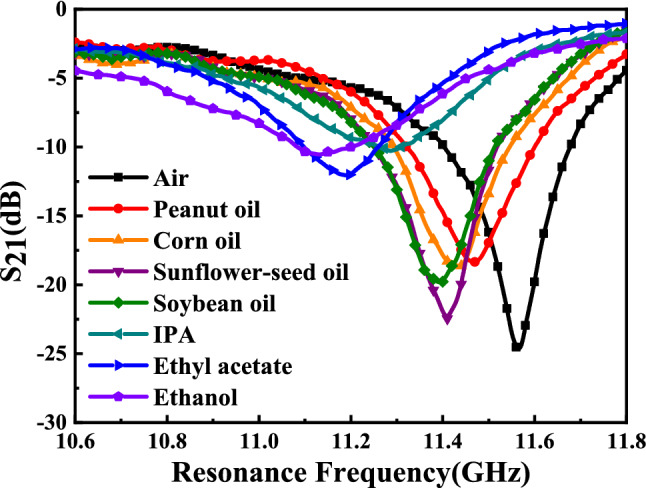
Measured transmission coefficient ${S}_{21}$ (dB) of sensor due to interaction with different organic liquids. Resonant frequencies of sensors due to interaction with Air, Peanut oil, Corn oil, Sunflower-seed oil, Soybean oil, IPA, Ethyl acetate, Ethanol are 11.575 GHz, 11.470 GHz, 11.420 GHz, 11.410 GHz, 11.390 GHz, 11.287 GHz, 11.200 GHz and 11.150 GHz, respectively.

The resonant peak at around 11.60 GHz is sensitive to the small change of dielectric environment, so we try to measure different liquids with low dielectric constant to verify the performance of the sensor. Different organic liquids that have a homogeneous dielectric distribution and high fluidity, such as peanut oil (*LuHua*), corn oil (*Longevity Flower*), sunflower seed oil (*Longevity Flower*) soybean oil (*Golden dragon fish*), IPA (*DongWu*), Ethyl acetate (*DongWu*), and ethanol (*Aladdin*) were chosen as MUTs. In order to minimize the impact of contamination and humidity from the previous test sample liquids, we washed the channel with detergent and brush firstly, then rinsed the channel repeatedly with alcohol solution and dried the remaining alcohol with a small hair dryer. Finally, after the sensor was laid flat for about 30 s to ensure that the alcohol evaporates adequately, the next liquid sample was dropped in the channels. When measuring the volatile liquid samples, we record the measured data quickly. Figure 14 shows the overall experiment platform for measuring different liquid samples. When measuring liquid samples, the sensor needs to be inverted so that one side of the microchannel is on top, which facilitates the loading of liquid samples. Each sample was measured about 15 times to ensure the reliability of the measured results. The measured results ${S}_{21}$ of different liquids sample are presented in Fig. 15 and the specific measured data is shown in Table 3. The carve of air is regarded as a reference signal, and the other liquids curves with different resonant frequency are measured transmission coefficient ${S}_{21}$. It’s obvious that there are different resonant frequencies when the channel with different samples, so the proposed sensor can be used for identifying different liquids with low permittivity.

**Table 3:** Measured results of the microwave sensor with different organic liquids.

Liquid samples	Literature ${\varepsilon }_{r}$	Resonant frequency (GHz)	Notch depth (dB)
Ethanol	7.02	11.150	− 10.5
Ethyl acetate	6.04	11.200	− 12.0
IPA	4.38	11.287	− 10.3
Soybean oil	2.99	11.390	− 19.6
Sunflower seed oil	2.75	11.410	− 22.5
Corn oil	2.63	11.420	− 18.6
Peanut oil	2.01	11.470	− 18.4
Air	1.00	11.575	− 24.7

### Simulation model of the T-shape microfluidic channel

**Figure 16:**
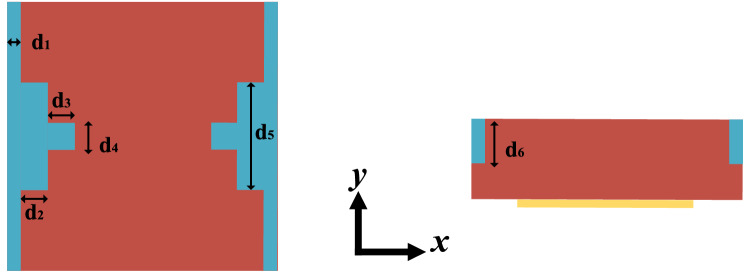
Simulation model of the proposed microwave sensor using CST with the sample liquids filling with the channels.

The measured results show that different liquid samples with low dielectric constant can be distinguished by the sensor. The resonance frequency and amplitude of the resonant peak can be separately used for calculating the real part and the imaginary part of the unknown liquids’ permittivity^19^. Considering that the attenuation of the patch antenna has a great impact on the measured amplitude, so we only can analyze the real part of the sample liquids’ permittivity. According to the actual situation of the liquids in the measurement, a relatively accurate model, which includes the microfluidic channel part, is built and shown in Fig. 16. The blue part of the model is the channel filled with different liquids. The geometrical parameters of T-shape channel shown in Fig. 16 were given in Table 4. By changing the dielectric constant of the liquids in the model, making the simulated resonant frequency fit the measured results as much as possible, then we can get a permittivity of the liquid and the obtained value is very close to the real dielectric constant of the liquid.

**Table 4:** Structure parameters of T-shape channel.

Parameter	Value [mm]
${{\varvec{d}}}_{1}$	0.5
${{\varvec{d}}}_{2}$	1.0
${{\varvec{d}}}_{3}$	1.0
${{\varvec{d}}}_{4}$	1.0
${{\varvec{d}}}_{5}$	4.0
${{\varvec{d}}}_{6}$	0.5

### Analysis of the measured results of different organic liquids

**Figure 17:**
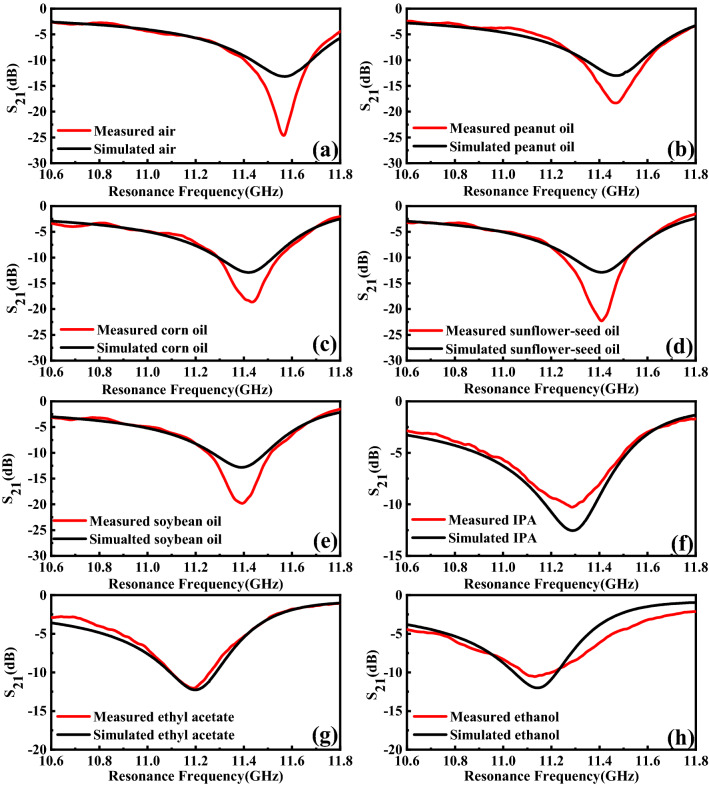
Simulated and measured resonant frequency of (**a**) Air (reference). (**b**) Peanut oil. (**c**) Corn oil. (**d**) Sunflower-seed oil. (**e**) Soybean oil. (**f**) IPA. (**g**) Ethyl acetate. (**h**) Ethanol.

**Figure 18:**
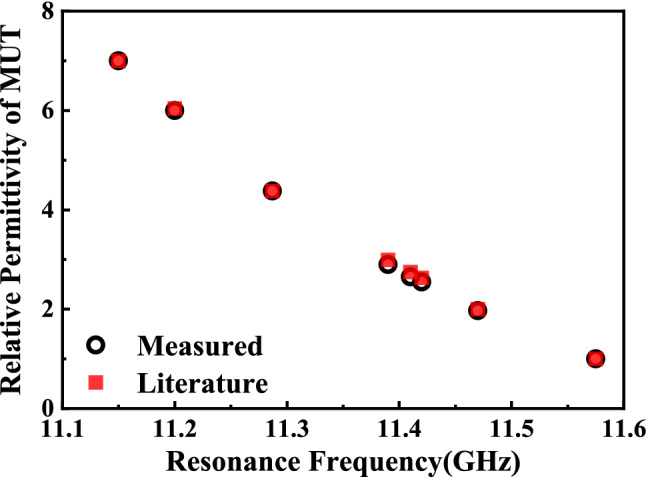
Comparison between the literature values of the relative permittivity from^5,16,17,20–25^ and the ones measured by using the designed sensor.

Using the T-shape channel model mentioned above, the relative permittivity of liquids can be obtained and the comparison of measured results and simulated results are shown in Fig. 17. The difference of amplitude is mainly caused by the fabrication tolerance, conductor, dielectric and radiation losses. The literature and measured dielectric constants of different organic liquid samples are tabulated in Table 5 which shows the maximum error of the measured results is 3.63%. As Fig. 18 shows, the measured ${\varepsilon }_{r}$ of peanut oil, corn oil, sunflower oil, soybean oil, IPA, ethyl acetate, and ethanol match well with those measured in the literature^5,7,20–24^, which indicates the reliability and accuracy of the measured results and the simulation model.

**Table 5:** Measured and simulated results of the microwave sensor with different organic liquids.

Liquid samples	Measured* f* (GHz)	Simulated *f* (GHz)	Literature ${\varepsilon }_{r}$	Measured ${\varepsilon }_{r}$	Measured error (%)
Ethanol	11.150	11.140	7.02	7.00	0.28
Ethyl acetate	11.200	11.198	6.04	6.00	0.66
IPA	11.287	11.288	4.38	4.38	0.00
Soybean oil	11.390	11.390	2.99	2.90	3.01
Sunflower-seed oil	11.410	11.410	2.75	2.65	3.63
Corn oil	11.420	11.420	2.63	2.55	3.04
Peanut oil	11.470	11.470	2.01	1.97	2.03
Air	11.575	11.568	1.00	1.00	0.00

### An empirical relationship between the dielectric constant and resonant frequency for liquids with low permittivity

Galindo-Romera et al. proposed a parabolic equation^20^ between resonant frequency *f* and dielectric constants ${\varepsilon }_{r}$ which can be used to estimate the relative permittivity of some other unknown liquids. The parabolic equation with three constant parameters is as follows:

5 $${f}_{r.MUT}={A}_{1}+{A}_{2}{\varepsilon }_{r}^{{{\prime}}}+{A}_{3}{{\varepsilon }_{r}^{{{\prime}}}}^{2}$$

Here, ${\varepsilon }_{r}^{{{\prime}}}$ is the relative permittivity of liquid sample. ${A}_{1}$, ${A}_{2}$, and ${A}_{3}$ are constant values. The reference MUT is air whose dielectric constant is 1. Considering that ${f}_{r.Air}$, the resonant frequency of sensor with empty channel, is a constant value. Based on reference^19^, Eq. (5) can be expanded with respect to $({\varepsilon }_{r}^{{{\prime}}}-1)$, as (6) shows:

6 $${f}_{r. MUT}=11.575+{A}_{2}\left({\varepsilon }_{r}^{{{\prime}}}-1\right)+{A}_{3}{({\varepsilon }_{r}^{{{\prime}}}-1)}^{2}$$

Based on the measured results of different liquids, the constant parameters ${A}_{1}$, ${A}_{2}$, and ${A}_{3}$ of (6) can be determined. The final parabolic Eq. (6) becomes

7 $${f}_{r. MUT}=11.575-0.10863\left({\varepsilon }_{r}^{{{\prime}}}-1\right)+0.00646{({\varepsilon }_{r}^{{{\prime}}}-1)}^{2}$$

The curve of the parabolic Eq. (7) is shown in Fig. 16. To calculate the relative permittivity of unknown liquids, the transcendental equation can be expressed as:

8 $${\varepsilon }_{r}^{{{\prime}}}=\frac{0.10863-\sqrt{0.0118-0.2584(11.575-{f}_{r. MUT})}}{0.01292}+1$$

**Figure 19:**
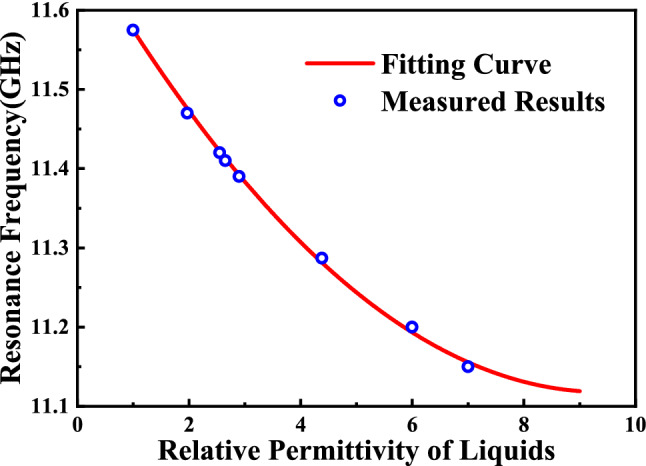
The carve of the parabolic Eq. (7) based on measured results.

Based on the measured resonant frequency, the transcendental equation can be used to estimate relative permittivity of unknown liquids with permittivity ranges from 1 to 9, as Fig. 19 shows. The literature permittivity, measured permittivity and the calculated permittivity of samples were compared in Fig. 19 which verified the reliability of the transcendental equation. And the calculated error in Table 6 show that the maximum calculated error of transcendental equation is 2.71%.

**Table 6:** Dielectric constant calculated using measured resonant frequency and transcendental Eq. (8).

Liquid samples	Measured ${\varepsilon }_{r}$	Calculated ${\varepsilon }_{r}$	Calculated error (%)
Ethanol	7.00	7.19	+ 2.71
Ethyl acetate	6.00	5.86	− 2.33
IPA	4.38	4.30	− 1.83
Soybean oil	2.90	2.92	− 2.34
Sunflower seed oil	2.65	2.69	+ 0.14
Corn oil	2.55	2.57	+ 0.78
Peanut oil	1.97	2.02	+ 2.54
Air	1.00	1.00	− 0.00

**Figure 20:**
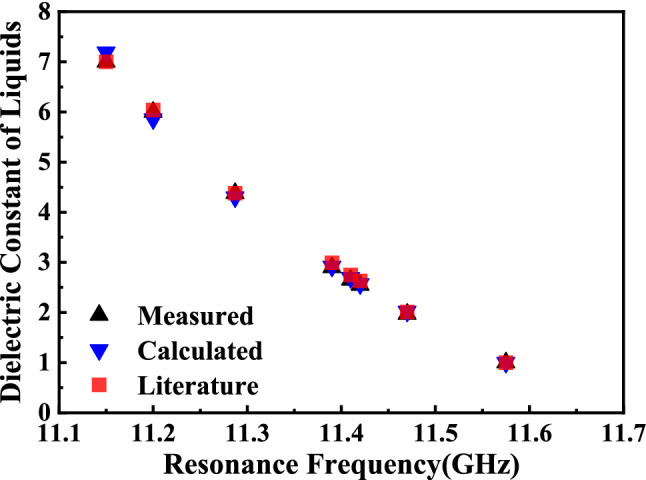
Comparison between the measured, calculated and literature values of dielectric constant for different liquids.

Figure 20 shows that the calculated and measured dielectric constants of different liquid samples measured in this paper agree well with those reported in the literature^5,7,20–24^, which indicates the reliability and accuracy of the transcendental equation to a certain extent. The estimated and measured values of samples’ dielectric constant are a little lower than the literature values, mainly for the reason that the dielectric constant of liquid will decrease with the increase of frequency^25^.

## Measurement for solid dielectric substrates

### Simulation and measurement of common solid dielectric materials

**Figure 21:**
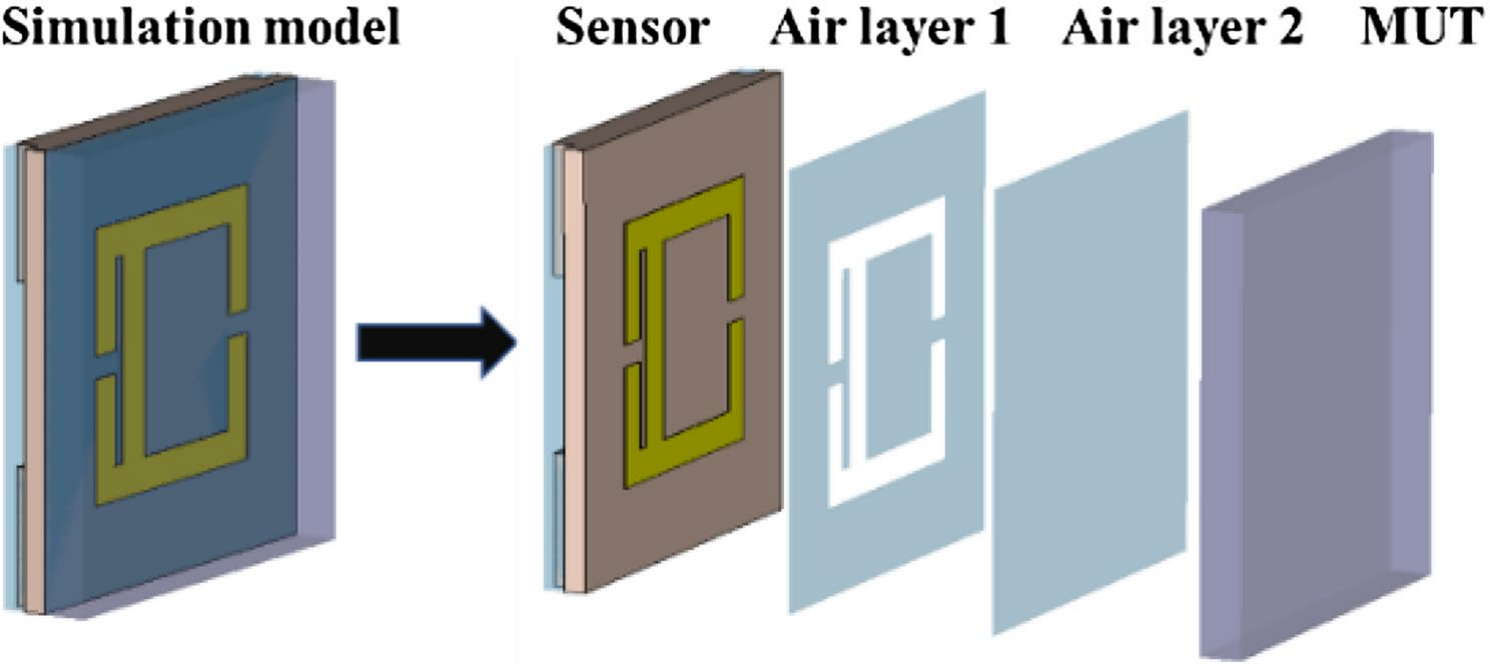
Simulation model and its profile chart which includes the air layer.

**Figure 22:**
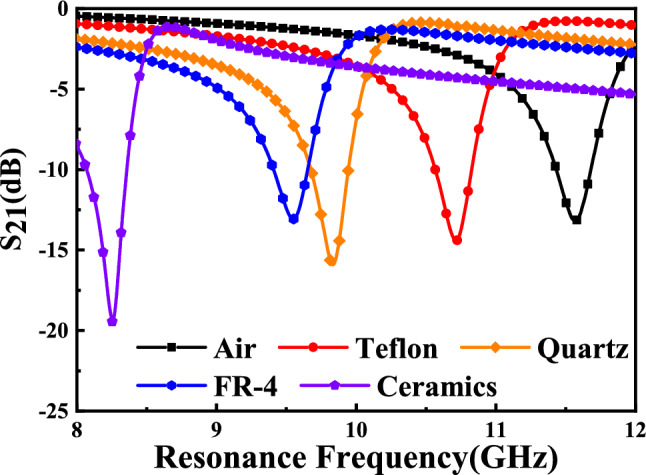
Simulated transmission coefficient ${S}_{21}$ (dB) of sensor due to interaction with different MUTs. Resonant frequencies of sensors due to interaction with Air, Teflon, Quartz, FR-4, and Ceramics are 11.57 GHz, 10.71 GHz, 9.84 GHz, 9.55 GHz, and 8.25 GHz, respectively.

Considering the actual measurement situation of the solids, a simulation model including air layers was built in CST. In the actual measurement, the slight bending of the MUTs resulted in the fact that the MUTs and the sensor did not fit tightly, so we added the air layer 2 to the model shown in Fig. 21 to ensure the accuracy of simulation results^12^. The thickness of air layer 1, air layer 2 and MUT are 0.03 mm, 0.02 mm and 1 mm, respectively. Common solid dielectric materials (Teflon, Quartz, FR-4, Ceramics) were simulated and the simulated results is shown in Fig. 22. The simulated results show that the sensor has the ability to distinguish different solid materials with high sensitivity and large frequency shift ∆ *f*.

**Figure 23:**
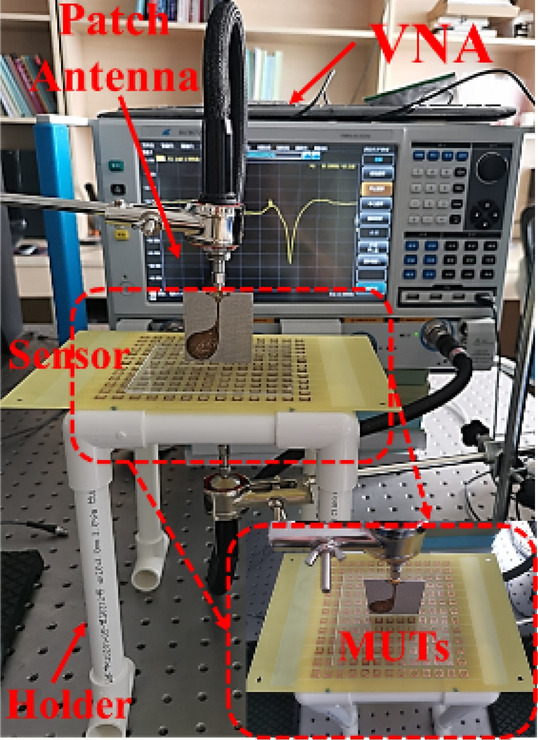
Photograph of the experiment platform for measuring different solid MUTs.

**Figure 24:**
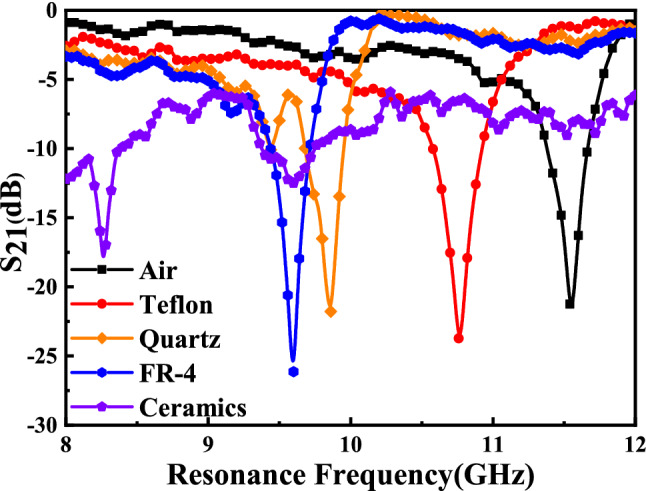
Measured transmission coefficient ${S}_{21}$ (dB) of sensor due to interaction with different MUTs. Resonant frequencies of sensors due to interaction with Air, Teflon, Quartz, FR-4, and Ceramics are 11.575 GHz, 10.760 GHz, 9.860 GHz, 9.600 GHz, and 8.260 GHz, respectively.

As Fig. 23 shows, a measurement platform was set up according to the schematic diagram of Fig. 12. When measuring different solid samples, the sensor needs to be squared so that one side of the array structure is at the top, which facilitates the placement of solid samples. The measured results of sensor with different MUTs were shown in Fig. 24 which are basically consistent with the simulated results.

### Analysis of the measured results of different solid materials

**Figure 25:**
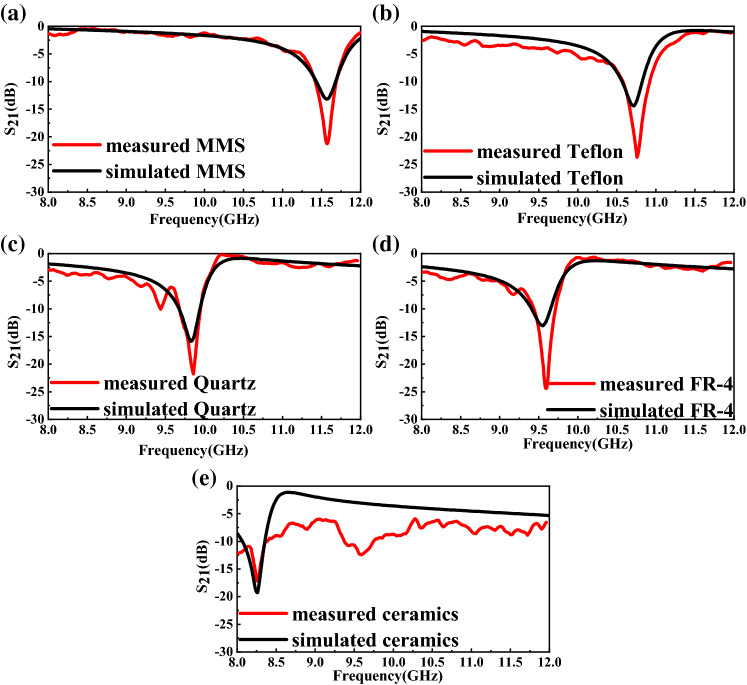
Comparison between simulated and measured transmission coefficient ${S}_{21}$ (dB) of (**a**) Air (reference). (**b**) Teflon. (**c**) Quartz. (**d**) FR-4. (**e**) Ceramics (Al_2_O_3_).

**Figure 26:**
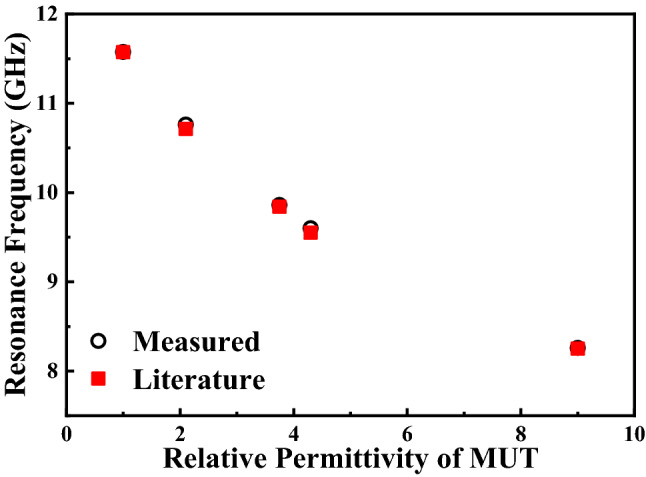
Comparison between the literature values of the relative permittivity from [9–12] and the ones measured using the design sensor.

The relative permittivity of Air, Teflon, Quartz, FR-4, and Ceramics (${\mathrm{Al}}_{2}{\mathrm{O}}_{3}$) are well known and shown in Table 7. The comparison between simulated and measured results is shown in Fig. 25 and Table 7 which verified the accuracy of the simulation model and the measured results. And the measured error in Table 7 shows that FR-4 has the maximum measured error with 0.523% and Teflon has the minimum measured error with 0.047%. The differences between simulated and measured resonant frequency are very small and can be attributed to fabrication tolerance and measurement errors. And the differences between simulated and measured amplitude are mainly caused by the fabrication tolerance, conductor, dielectric and radiation losses. The irregularity of the measured carve is mainly caused by the heterogeneity of the MUTs and dielectric and radiation losses. Figure 26 shows that the measured relative permittivity of MUTs match well with the literature values reported in references^9–12^, which indicates the accuracy of the measured results and the reliability of the sensor proposed in this paper.

**Table 7:** Comparison between simulated and measured results of sensors for different MUTs.

MUTs	$\upvarepsilon$	Simulated *f* (GHz)	Measured *f* (GHz)	Measured error (%)
Air	1	11.570	11.575	+ 0.043
Teflon	2.1	10.710	10.760	+ 0.047
Quartz	3.75	9.840	9.860	+ 0.203
FR-4	4.3	9.550	9.600	+ 0.523
Ceramics (${\mathrm{Al}}_{2}{\mathrm{O}}_{3}$)	9.0	8.250	8.260	+ 0.121

### An empirical relationship between the dielectric constant and resonant frequency for solids with low permittivity

In this paper, the fitting equation for the whole sensor are formulated with fitting parameters using measured results. The resonant frequencies of the sensor very when material under test (MUT) is placed on the sensor^26^. The variation in resonant frequency can be expressed by the following equation:

9 $${f}_{r.MUT}={f}_{r.Air}\sqrt{\frac{{\varepsilon }_{eff,Air}}{{\varepsilon }_{eff,MUT}}}$$

where ${f}_{r.MUT}$ and ${f}_{r.Air}$ are resonant frequencies of sensor with and without MUT, respectively. And ${\varepsilon }_{eff,Air}$ and ${\varepsilon }_{eff,MUT}$ are effective permittivity of air and MUTs, respectively. Equation (10) shows the relationship between relative permittivity of MUT and the resonant frequency of sensor due to interaction with MUT. And the relationship shows that the resonant frequency is decreasing by the increasing the relative permittivity of MUT. In reference^26^, a parabolic equation between relative permittivity of MUTs and the resonant frequency of sensor is established. It’s shown in the following equation:

10 $${f}_{r.MUT}={A}_{1}+{A}_{2}{\varepsilon }_{r}^{{{\prime}}}+{A}_{3}{{\varepsilon }_{r}^{{{\prime}}}}^{2}$$

Here, ${\varepsilon }_{r}^{{{\prime}}}$ is relative permittivity of MUT. ${A}_{1}$, ${A}_{2}$, and ${A}_{3}$ are constant values. The reference MUT is air whose dielectric constant is 1. Considering that the resonant frequency of sensor without MUT ${f}_{r.Air}$ is a constant value and based on reference^19^, Eq. (10) can be expanded with respect to $({\varepsilon }_{r}^{{{\prime}}}-1)$, as Eq. (11) shows:

11 $${f}_{r. MUT}={A}_{1}+{A}_{2}\left({\varepsilon }_{r}^{{{\prime}}}-1\right)+{A}_{3}{({\varepsilon }_{r}^{{{\prime}}}-1)}^{2}$$

Based on the measured results of materials (Air, Teflon, Quartz and Ceramics), the constant parameters ${A}_{1}$,${A}_{2}$, and ${A}_{3}$ of (11) can be determined. Then, Eq. (11) becomes:

12 $${f}_{r. MUT}=11.575-0.74629\left({\varepsilon }_{r}^{{{\prime}}}-1\right)+0.04152{({\varepsilon }_{r}^{{{\prime}}}-1)}^{2}$$

Materials (FR-4) are stand dielectric substrate for which dielectric constant is well known. We used (12), fitted based on the measured results of other MUTs, to estimate the relative permittivity of FR-4 to test the reliability of this empirical relationship. The ${\varepsilon }_{r}^{{{\prime}}}$ value obtained based on measured resonant frequency is 4.23 which is closed to the relative permittivity 4.3. It’s clearly that Eq. (12) is fairly reliable for predicting the dielectric constants of known MUTs based on measured ${f}_{r. MUT}$. To calculate the relative permittivity of known MUT, Eq. (12) can be express as:

13 $${\varepsilon }_{r}^{{\prime}}=\frac{0.74629-\sqrt{0.55695-0.16608(11.575-{f}_{r. MUT})}}{0.08304}+1$$

**Figure 27:**
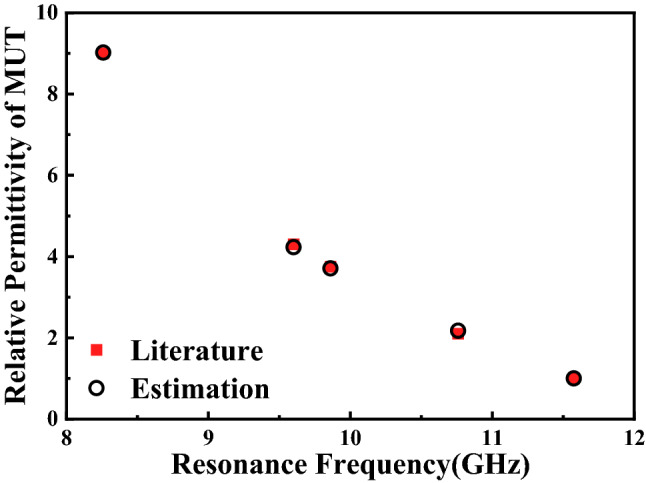
Comparison between the literature values of the relative permittivity from reference^9–12^ and the ones estimated using the transcendental Eq. (13).

Equation (13) can be used to calculate relative permittivity of known MUTs. In order to check the reliability and validity of the simulation model and (13), the relative permittivity of different MUTs are calculated based on the measured ${f}_{r. MUT}$ using proposed sensor and are tabulated in Table 8. The estimated error in Table 8 shows the maximum estimated error of transcendental Eq. (13) is 3.3%. And Fig. 27 shows the calculated relative permittivity agree well with the literature values. The reliability of calculated ${\varepsilon }_{r}^{{{\prime}}}$ shows that the sensor has the ability to identify different MUTs and predict their dielectric constant within a certain range of accuracy.

**Table 8:** Estimated relative permittivity using measured results and Eq. (13).

Material under test (MUT)	Relative permittivity (${\varepsilon }_{r}^{{\prime}}$)	Estimated ${\varepsilon }_{r}^{{\prime}}$ based on (13)	Estimated error (%)
Air	1	1.00	+ 0.0
Teflon	2.1	2.17	+ 3.3
Quartz	3.75	3.71	− 1.1
FR-4	4.3	4.23	− 1.6
Ceramics	9	9.02	+ 0.2

## Performance comparison

The microwave sensor proposed in this paper can be used not only for identifying organic liquids but also for distinguishing solid substrates. So as to place the present work in context, the performance of the proposed sensor is compared with microwave sensors for liquids and microwave sensors for solids reported in the prevailing literature. Moreover, to make a fair comparison of the sensitivity between the proposed sensor and other microwave sensors, we use the mean sensitivity S defined in reference^27^ and shown in (14):

14 $$S= \left(\frac{{f}_{{\varepsilon }_{r2}}-{f}_{{\varepsilon }_{r1}}}{{f}_{0}({\varepsilon }_{r2}-{\varepsilon }_{r1})} \right)\times 100$$

### Comparison with prevailing sensors for liquids

Table 9 presents the performance characteristics of several conventional microwave sensors with various configurations, resonant frequencies, and excitation sources, etc. Most of conventional meta-atom sensors excited by microstrip line are used for liquids whose permittivity ranges from 9 to 80. The proposed sensor, excited by a pair of antennas, is designed for liquids with low permittivity which complements the detection range of traditional sensors. Moreover, based on the measured results and mean sensitivity ***S*** defined in reference^27^, Table 9 shows that the proposed metamaterial-inspired sensor can distinguish different liquids whose permittivity ranges from 1 to 9 with high mean sensitivity.

**Table 9:** Comparison of the proposed sensor with other microwave sensor for liquids testing.

Ref.	Sensor	${f}_{res}$[GHz]	Contact	Excitation source	Permittivity range studied	$\Delta f$/$\Delta\upvarepsilon$ (MHz)	Mean sensitivity *S* $\left(\frac{{{\varvec{f}}}_{{{\varvec{\varepsilon}}}_{{\varvec{r}}2}}-{{\varvec{f}}}_{{{\varvec{\varepsilon}}}_{{\varvec{r}}1}}}{{{\varvec{f}}}_{0}({{\varvec{\varepsilon}}}_{{\varvec{r}}2}-{{\varvec{\varepsilon}}}_{{\varvec{r}}1})}\right)\times 100$
^3^	CSRR	2.4	No	Microstrip line	9–80	4.30	0.179
^4^	CSRR	2.1	No	Microstrip line	9–80	1.72	0.082
^5^	CCSR	2.4	Yes	Microstrip line	11–60	10.08	0.420
^8^	Circular CSRRs	2.4	No	Microstrip line	9–79	0.05	0.002
^13^	SRR	3	No	Antenna	13–70	1.05	0.035
^27^	MTM sensor	2.6	No	Microstrip line	1–140	7.02	0.27
^28^	$\lambda /2$	2.4	Yes	Microstrip line	7.5–22	1.68	0.070
Proposed	AESRR metamaterial	11.5	Yes	Antenna	1–9	70.38	0.612

### Comparison with prevailing sensors for solids

Table 10 presents the performance characteristics of several conventional microwave sensors with various configurations, excitation sources, permittivity range studied and frequency shift *∆f*, etc. Many conventional meta-atom sensors excited by microstrip line have been reported to be used for distinguishing different dielectric materials and predicting their permittivity. But conventional meta-atom sensors have a lot of room for improvement in terms of frequency shift *∆f* and sensitivity. Moreover, Table 8 shows that the proposed metamaterial-inspired sensor, excited by antenna, can distinguish different solid dielectric materials with bigger frequency shift *∆f* and higher mean sensitivity.

**Table 10:** Comparison of the proposed sensor with other microwave sensor for solids testing.

Ref.	Sensor	${f}_{res}$[GHz]	Excitation source	Permittivity range studied	$\Delta f$/$\Delta\upvarepsilon$ (GHz)	Mean sensitivity *S* $(\frac{{{\varvec{f}}}_{{{\varvec{\varepsilon}}}_{{\varvec{r}}2}}-{{\varvec{f}}}_{{{\varvec{\varepsilon}}}_{{\varvec{r}}1}}}{{{\varvec{f}}}_{0}({{\varvec{\varepsilon}}}_{{\varvec{r}}2}-{{\varvec{\varepsilon}}}_{{\varvec{r}}1})})\times 100$
^9^	CCSR	2.3	Microstrip line	1–7	0.06	3.45
^10^	SRR	5.0	Microstrip line	1–9	0.12	2.30
^10^	$\mathrm{SRR}$	10.0	Microstrip line	1–9	0.25	2.50
^11^	CCSRs	1.0	Microstrip line	1–4.3	0.06	3.57
^12^	CSRR	2.7	Microstrip line	2.2–10.2	0.07	2.73
Proposed	AESRR metamaterial	11.5	Antenna	1–9	0.415	3.59

## Conclusion

A high-sensitivity microwave metamaterial-inspired sensor, based on a 13 × 13 arrays of Asymmetric Electric Split-Ring Resonator (AESRR), is presented for the permittivity characterization of organic liquids and solid dielectric substrates with low permittivity. Excited by a pair of patch antennas, the sensor exhibits strong electric field in the gaps of AESRR which allows the sensor is sensitive to the change of dielectric environment. T-shape channels were integrated to the sensor by grooving in the substrate to improve the integration and enable the feasibility of liquids detection.

During the measurement session, seven organic liquids and four solid dielectric substrates were chosen as MUTs and the measured results match well with the simulated results which verified the reliability of sensor. Based on the fabricated sensor and actual measurement environment, simulation models of measuring liquids and measuring solids were built in CST, respectively. Moreover, two transcendental equations, derived from the measured results, are proposed to predict the relative permittivity of liquid samples and solid materials, respectively. And the estimated values of relative permittivity are in good agreement with the literature values showing the accuracy of transcendental equations. The proposed sensor and these two transcendental equations are mainly suitable for low permittivity liquid samples and low permittivity solid samples whose permittivity ranges from 1 to 9.

Compared to prevailing conventional meta-atom microwave sensors excited by microstrip line, the proposed sensor can distinguish not only liquids but also solid dielectric materials with bigger frequency shift $\Delta f$ and higher sensitivity. This sensor has many advantages, such as low-cost, real-time, high-sensitivity, and high-robustness. Most importantly, it applies to the permittivity characterization of organic liquids as well as solid dielectric substrates—a wider range of applications, which makes the sensor an attractive choice to be implemented in a lab-on-a-chip sensor system in the microwave band.

Future work will focus on increasing the sensitivity of sensor and reducing sensor size and reducing the volume/area of MUTs.
